# Extra-islet cells expressing insulin or glucagon in the pancreas of young organ donors

**DOI:** 10.1007/s00592-024-02295-0

**Published:** 2024-06-18

**Authors:** Louise Granlund, Olle Korsgren, Oskar Skog, Marcus Lundberg

**Affiliations:** 1 Department of Immunology, Genetics and Pathology, Uppsala University, Uppsala, Sweden; 2 Department of Clinical Chemistry and Transfusion Medicine, Institute of Biomedicine, University of Gothenburg, Gothenburg, Sweden

**Keywords:** Extra-islet cells, Type 1 diabetes, Human pancreas, Proliferation, Transcription factors

## Abstract

**Aims:**

The existence of insulin- or glucagon-expressing extra-islet endocrine cells scattered in the pancreas is well-known, but they have been sparsely characterized. The aim of this study was to examine their density, distribution, transcription-factor expression, and mitotic activity in young non-diabetic subjects.

**Methods:**

Multispectral imaging was used to examine PDX1, ARX, Ki67, insulin and glucagon in extra-islet endocrine cells in pancreatic tissue from organ donors aged 1–25 years.

**Results:**

Extra-islet insulin- or glucagon-positive cells were frequent in all donors (median 17.3 and 22.9 cells/mm^2^ respectively), with an insulin:glucagon cell ratio of 0.9. The density was similar regardless of age. PDX1 localized mainly to insulin-, and ARX mainly to glucagon-positive cells but, interestingly, many of the cells were negative for both transcription factors. Double-hormone-positive cells were rare but found in all age groups, as were insulin-positive cells expressing ARX and glucagon-positive cells expressing PDX1. Extra-islet endocrine cells with Ki67 expression were present but rare (0–2%) in all age groups.

**Conclusions:**

Extra-islet endocrine cells are more frequent than islets. The preserved extra-islet cell density during pancreas volume-expansion from childhood- to adulthood indicates that new cells are formed, possibly from replication as cells with mitotic activity were discovered. The lack of transcription-factor expression in many cells indicates that they are immature, newly formed or plastic. This, together with the mitotic activity, suggests that these cells could play an important role in the expansion of beta-cell mass in situations of increasing demand, or in the turnover of the endocrine cell population.

**Supplementary Information:**

The online version contains supplementary material available at 10.1007/s00592-024-02295-0.

## Introduction

In the pancreas, the endocrine cells arranged in islets of Langerhans have been studied extensively [1]. However, there are also extra-islet endocrine cells scattered as single cells, or in groups of a few cells. These cells are sparsely characterized but may have a role in the development and turnover of the endocrine cells in the pancreas.

Post-natal human β-cell proliferation is highest during infancy and declines until approximately 5 years of age, whereafter it stabilizes [2]. The beta-cell mass thereafter continues to expand until adulthood [3]. The beta-cell mass varies among individuals [4], and is dynamic, the latter being especially apparent in certain settings. For example, in human pregnancy, the beta-cell fraction of the pancreatic area is increased by approximately 40%, derived mainly from an increased number of small islets [5]. Additionally, in obese human subjects beta-cell mass is increased approximately 50% due to an elevated beta-cell number [4], and in insulin-resistant non-diabetic subjects there is an increased islet size and islet density [6]. Mechanisms leading to increased beta-cell mass are (1) replication of already existing beta cells [3, 7–9], and (2) beta-cell neogenesis [10–13]. In addition to settings where an increased demand causes an increased beta-cell mass, there is also an ongoing turnover of beta cells in adulthood [9], although the extent and mechanisms involved are debated and need to be clarified [9, 14].

The frequency of scattered insulin-positive cells has been examined before [15–17]. In 1998, Bouwens and Pipeleers reported that 15% of all beta cells were found in units of ≤ 3 beta cells without associated alpha-, delta-, or pancreatic polypeptide-cells [18]. These were smaller in size compared to beta cells in islets, were mainly located in or along ductules from which they appeared to bud from and were negative for the mitosis marker Ki67. More recently it has been shown that the number of replicating endocrine cells in the pancreas is likely underestimated due to methodological factors [7]. We hypothesized that the extra-islet insulin- and/or glucagon-positive single-cells scattered in the exocrine compartment have a mitotic activity and could be an important cell type in the generation of new islets. The aim of the current study was to describe the extra-islet insulin- and glucagon-positive cell populations in more detail in well-preserved pancreatic tissue obtained from heart-beating organ donors, by determining their density, distribution, mitotic activity, as well as the expression of alpha- and beta-cell transcription factors ARX and PDX1. By utilizing multispectral imaging, all these could be examined simultaneously in the same section. As the beta-cell mass expansion is prominent until young adulthood [3, 19], and the density, as well as the expression of these markers, could vary by age, we included donors aged 1–25 years old in the study.

## Material and methods

### Ethics

Consent for organ donation (for clinical transplantation and for use in research) was obtained via online database (https://www.socialstyrelsen.se/en/apply-and-register/register/join-the-swedish-national-donor-register/) or verbally from the deceased’s next of kin by the attending physician and documented in the medical records of the deceased in accordance with Swedish law and as approved by the Swedish Ethical Review Authority (Dnr 2023-01845-01). All tissue included in the study was procured, stored and analyzed as approved by the Regional Ethics Committee in Uppsala (Dnr: 2015/444).

### Human pancreatic samples

The study was conducted using formalin-fixed and paraffin-embedded (FFPE) tissue samples from deceased organ donors procured within the Nordic Network for Islet Transplantation. Donors from three age groups (0–10, 11–18, and 19–25) were included in the study. Sections from the tail region of the pancreas were chosen for consistency and were available from six donors 0–10, seven donors 11–18 and nine donors 19–25 years old. However, one donor in the age group 19–25 years old had to be excluded due to insufficient tissue quality. The characteristics of all donors included are presented in Table 1. The tissue samples included in the study were collected between the years 2009 and 2018.

**Table 1:** Donor characteristics

Donor no	Age, years	BMI, kg/m^2^		Sex	HbA1c, %
A1	1	–	M		NA
A2	3	18.1	M		5.4
A3	5	21.9	F		NA
A4	5	13.9	M		5.2
A5	8	20.4	M		5.2
A6	10	17.3	F		NA
B1	12	14.9	M		5.4
B2	12	18.1	F		5.6
B3	13	19.7	M		5.2
B4	13	30.5	F		4.6
B5	14	25.5	F		5.5
B6	16	20.2	F		5.4
B7	17	28.9	F		NA
C1	19	21.0	M		5.3
C2	20	24.6	M		NA
C3	20	27.8	M		5.8
C4	21	21.1	M		5.2
C5	21	28.0	M		5.7
C6	24	25.3	F		5.9
C7	24	33.0	M		5.9
C8	25	22.9	M		5.9

*Donor No.* donor number, *Age* age of donor at the time of death, *BMI* body mass index, HbA1c in %. *NA* not available

### Multiplex staining of extra-islet cells

Concentrations and incubation times for the primary and secondary antibodies were optimized using FFPE tissue samples and standard immunohistochemistry (IHC). Primary and HRP-conjugated secondary antibodies (Supplementary table S1) were added followed by colourimetric detection using 3,3’-Diaminobenzidine (DAB). The sections were then counterstained with hematoxylin (Histolab), mounted under coverslips and visualized using a light microscope. The concentration and incubation time were optimised for each antibody separately on a section containing the target antigen, and on a section where the primary antibody was omitted. The protocol was then used with the Autostainer BOND RX System from Leica Biosystems (cat. nr. 21.2821, Wetzlar, Germany), where the concentrations and incubation times for the opals were further optimized. The multiplex staining protocol was then used on the tissue samples selected for the study.

The tissue samples were sectioned (6 µm) using a Microm HM355S (Thermo Fisher Scientific, Waltham, Massachusetts). One section per donor was used for multiplex staining of insulin, glucagon, PDX1, ARX and Ki67 using the Opal 6-Plex Detection Kit (cat. nr. NEL821001KT, Akoya Biosciences, Marlborough, Massachusetts) with the Autostainer. Briefly, all reagents were prepared and diluted in the BOND Titration Container Inserts placed in the BOND Titration Containers (cat. nr. OPT9049, Leica Biosystems). Dilutions were calculated based on the dead volume of the container of 300 µl, and a consumption of 150 µl per slide. The concentration of the primary and secondary antibodies as well as the Opals used are displayed in Supplementary table S1. The antibodies were diluted in the blocking buffer and the Opals in the amplification diluent provided in the Opal 6-Plex Detection Kit. Staining was performed in the following order: PDX1, INS, GCG, ARX and finally Ki67. Visualization was done with Opal 520, 570, 620, 690 and 480 respectively and the sections were counterstained with DAPI, diluted in TBST, provided in the kit. The tissue sections and BOND Titration containers containing all reagents were placed in the autostainer and the staining protocol, including deparaffinisation, was run overnight. The protocol for the autostainer can be found in the supplementary material. Six tissue sections were stained simultaneously, as this maximized the volume that the BOND titration container inserts could hold (6 ml) for the blocking buffer. After the autostainer had finished the staining, the slides were mounted manually using ProLong Diamond Antifade mounting medium (cat. nr. P36961, Thermo Fisher Scientific).

### Scanning and analysis of extra-islet cells

The slides were scanned at 20 × magnification with 0.05 µm pixel resolution and whole tissue-slide images were obtained using the Vectra Polaris multispectral imaging and whole slide scanning system (recently rebranded to *PhenoImager*, Akoya Biosciences). Exposure times were decided based on the average intensities in several areas in a number of sections, and the same exposure times were then used to scan all slides.

The obtained whole tissue slide images were analysed using the software Qupath (0.3.2). The annotations were done by a blinded investigator, not knowing which age category the donor belonged to, and lobes of the tissue sections were selected at random for analysis. A total of 200 extra islet cells were manually marked and named using the polygon tool in Qupath, and annotated according to the expression profile of that specific cell. Cells positive for ARX only or ARX and PDX1, without any hormone, were annotated as well, but no further analysis was performed on these cells. Single extra-islet cells, as well as small groups of up to 4 cells in direct contact with each other, interspersed in the exocrine parenchyma were annotated with regard to their phenotype. Their localisation was also noted and defined as: (1) peri-islet: being close to an islet (distance 0–3 cells) but outside of the islet perimeter, (2) peri-ductal: being close to a duct (distance 0–3 cells) but not within the ductal epithelium, and (3) intra-acinar: surrounded by acinar tissue and not being defined as peri-islet or peri-ductal. As no staining of ducts was performed, the extra-islet cells defined as being peri-ductal were restricted to extra-islet cells found adjacent to larger ducts that were morphologically distinguishable within the exocrine parenchyma. When several single cells and/or groups of 2–4 cells located close together in a cluster (but not in direct contact with each other) were found, that was also noted. A median of 57% of the annotated cells were present as single cells, 22% were found in groups of 2 cells, and the remaining in groups of 3–4 cells. When a total of 200 cells had been annotated per donor, the area of the analysed part of the tissue section was measured, using the polygon tool in Qupath.

### Immunofluorescent staining and analysis of islets

To validate the sensitivity in the multiplex stainings, immunofluorescent staining (IF) using the same primary antibodies as for the multiplex staining was performed, as this allowed the use of z-stacks to evaluate each islet cell. An FFPE tissue sample from a donor included in the study (donor no C1) was sectioned into 6 µm sections. The sections were boiled in pH 6 and blocked either for 15 min using the Antibody Diluent/Block (cat. nr. ARD1001EA) included in the Opal 6-Plex Detection Kit (insulin and PDX1 staining), or for 30 min using 5% donkey serum (glucagon and ARX staining). The sections were stained for either insulin and PDX1 or glucagon and ARX. For the insulin and PDX1 staining; the PDX1 antibody was diluted in the ready-to-use insulin antibody, and incubated for 1 h. The sections were washed 2 × 5 min in TBST, after which the secondary antibody to PDX1, AF488 rabbit-anti-goat, was added and incubated for 1 h (Supp. table S1). The sections were thoroughly washed 3 × 5 min in TBST, and the secondary antibody to insulin, AF647 goat-anti-guinea pig, was added and incubated for 1 h (Supp. table S1). The sections were washed 2 × 5 min in TBST, and 2 × 5 min in TBS. Nuclei were stained with 500 nM SYTOX Orange nucleic acid stain (10 min), the sections were washed 2 × 5 min in TBS and then mounted with Fluorescence Mounting Medium (Dako, cat. nr. S3023). For the glucagon and ARX staining; the two antibodies were diluted in 5% donkey serum, and incubated for 1 h. The sections were washed 2 × 5 min in TBST, after which the two secondary antibodies (A488 donkey-anti-rabbit and AF647 donkey-anti-sheep), were added and incubated for 1 h (supp. table S1). The sections were washed 2 × 5 min in TBST, and 2 × 5 min in TBS. Nuclei were stained and the sections mounted as described above.

The sections were imaged at 40× magnification and z-stacks were taken on 15 islets using the widefield microscope Zeiss AxioImager M2 together with an AxioCamMRM. The obtained z-stacks were deconvoluted using the Huygens Professional (20.04—Scientific Volume Imaging B.V.) software, and the final images were analysed in Qupath (0.3.2). The islet cells were annotated with regard to insulin and PDX1 expression, or glucagon and ARX expression. In total, 601 insulin + PDX1 ± cells and 1056 glucagon + ARX ± cells were annotated.

### Statistics

GraphPad Prism 9.5.0 (730) was used for visualization of the data and statistical analysis. To examine changes in insulin and glucagon density related to age simple linear regression was used. The Kruskal–Wallis test was used to compare the number of Ki67-positive cells between the donor groups.

## Results

### Both insulin- and glucagon-positive extra-islet cells were frequent with a limited change correlated to age

**Fig. 1:**
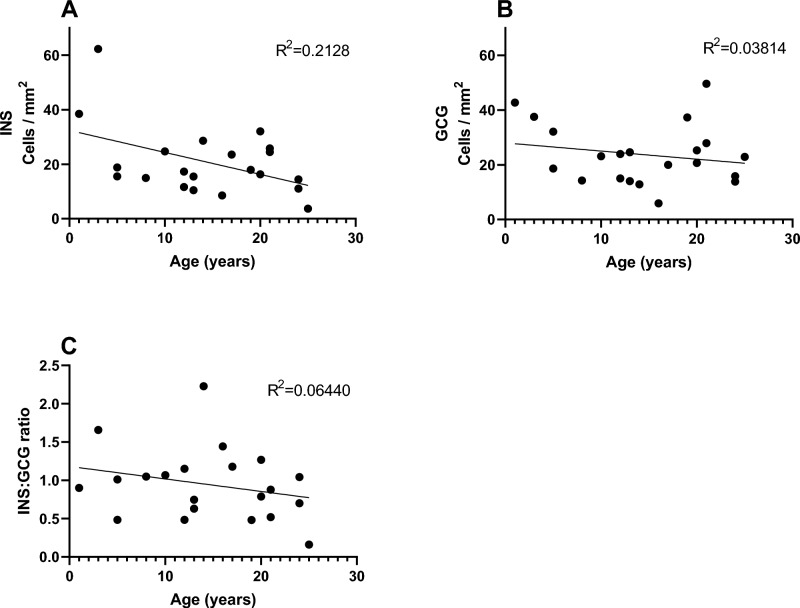
The density of extra-islet insulin- and glucagon-positive cells in relation to donor age. The density of insulin-positive cells (INS) per mm^2^ (**A**). The density of glucagon-positive cells (GCG) per mm^2^ (**B**). The ratio of insulin- to glucagon-positive cells (**C**). Each dot represents an individual donor

The median density of extra-islet insulin- and glucagon-positive cells were 17.3 and 22.9 cells/mm^2^, respectively, with a median ratio of insulin- to glucagon-positive cells of 0.9. The density of endocrine extra-islet cells was relatively constant over the age span, although a reduction in the density of insulin-positive cells correlated to age (*p* = 0.035, Fig. 1A) was seen when all donors were analysed. The two youngest donors (1 and 3 years old) were outliers with clearly higher insulin-cell density than the remaining 19 donors, and when the analysis included only donors ≥ 5 years old, no change in density of insulin-positive cells to age could be determined (*p* = 0.806). There was no change in the density of glucagon-positive cells (Fig. 1B) or insulin to glucagon-cell ratio over the age span (Fig. 1C). A median of 28% of the extra-islet endocrine cells was found in the peri-islet area and 1.5% in the peri-ductal area, but the majority (64%) were intra-acinar, not close to islet or ductal tissue (Supplementary figure S1).

### Many of the extra-islet insulin- and glucagon-positive cells in all age groups lacked the expression of PDX1 and ARX

**Fig. 2:**
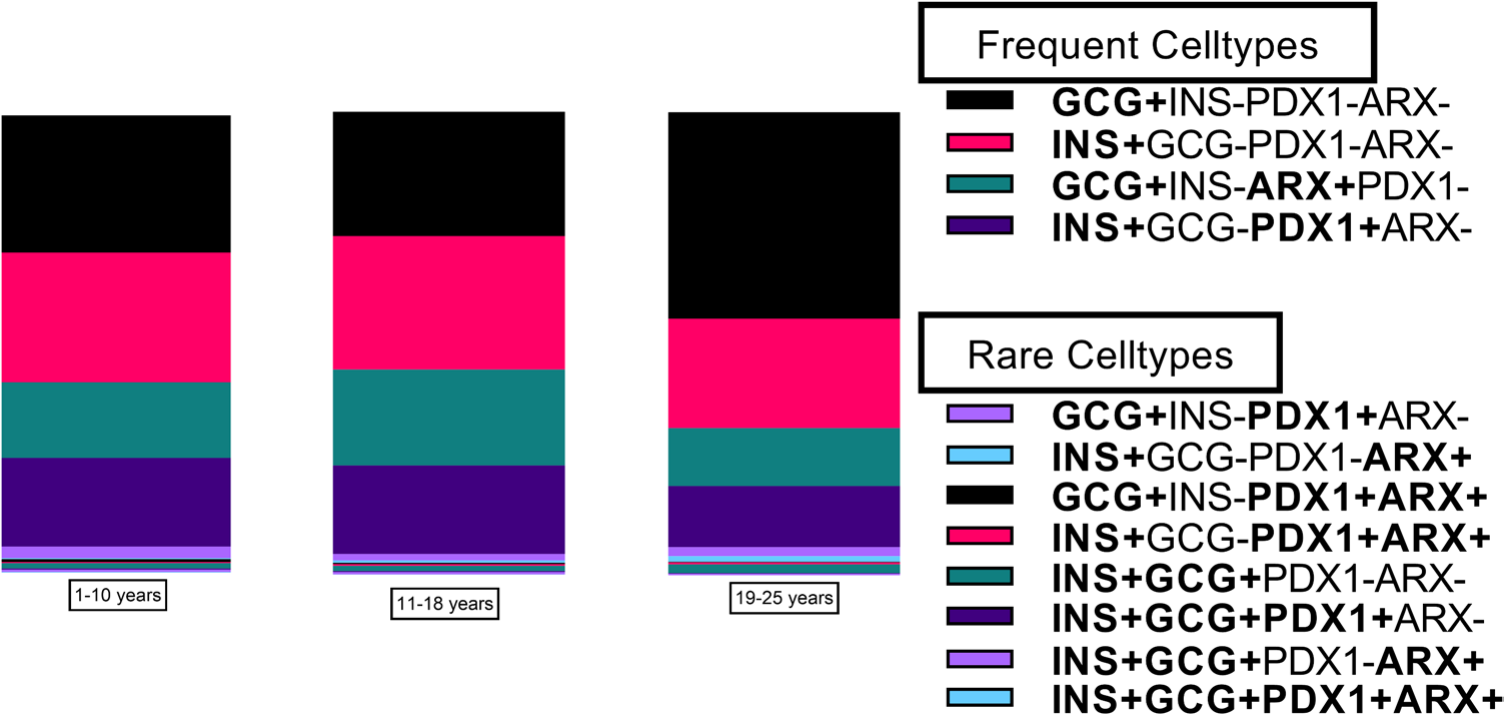
The proportion of extra-islet cell phenotypes in the three age groups. 200 cells were annotated in each donor. Except for cells only positive for ARX, or co-positive for only PDX1 and ARX, the proportion (%) of all remaining phenotypes based on combinations of insulin, glucagon, PDX1 and ARX in each age group is illustrated

**Fig. 3:**
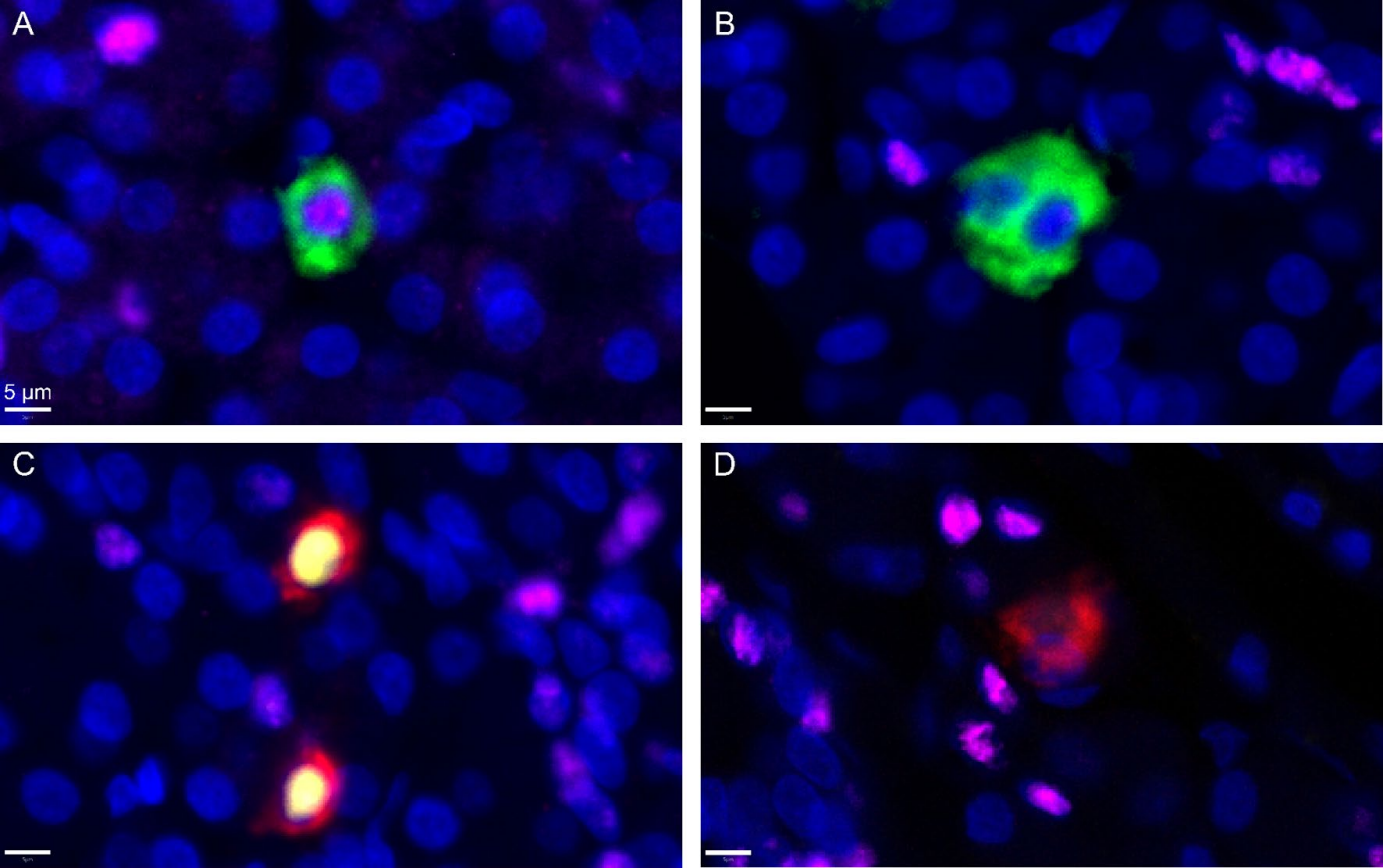
Representative images of the four most frequent cell types. Cells were stained for insulin (green), glucagon (red), PDX1 (magenta), ARX (yellow) and nuclei (DAPI, blue). **A** A cell positive for insulin and PDX1. **B** Two cells positive for insulin without PDX1. **C** Two cells positive for glucagon and ARX. D) A cell positive for glucagon without ARX. Scale bar = 5 µm  (Color figure online)

PDX1 localized mainly to insulin-positive cells, and ARX mainly to glucagon-positive cells but, interestingly, about half of the insulin- or glucagon-positive cells were negative for both transcription factors (Figs. 2, 3). However, in islets, the proportion of insulin-positive cells co-positive for PDX1 was 94% and glucagon-positive cells co-positive for ARX was 93% (representative images in Supplementary figure S3). Double-hormone-positive extra-islet cells were rare but found in all age groups, as were insulin-positive cells expressing ARX and glucagon-positive cells expressing PDX1. The proportion of the different cell phenotypes was similar independent of age. The proportion of all cell phenotypes in all donors is provided in Supplementary table S2, and representative images of rare cell types are provided in Supplementary figure S2. No obvious difference in phenotype depending on localisation was observed.

### Ki67-positive nuclei were found in some extra-islet endocrine cells in all age-groups

**Fig. 4:**
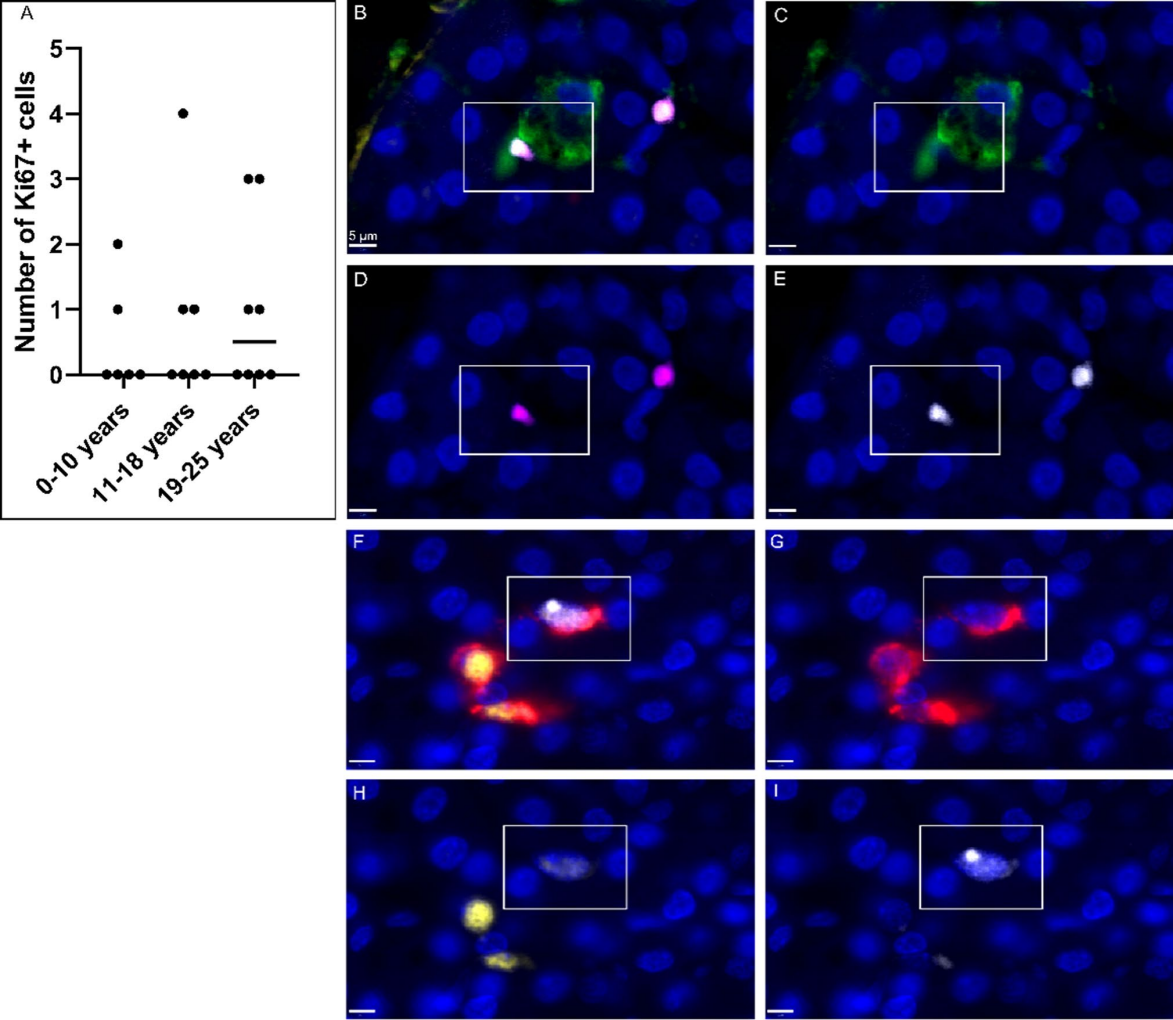
Ki67-positive extra-islet endocrine cells. **A** The number of Ki67-positive cells, among the 200 annotated cells per donor, in each age group. Each dot represents an individual donor. No significant difference could be determined between the different age groups (*p* = 0.8018). The lines illustrate the median. **B**–**I** Cells were stained for insulin (green), glucagon (red), PDX1 (magenta), ARX (yellow), Ki67 (white) and nuclei (DAPI, blue). **B**–**E** Representative image of a cell positive for insulin, PDX1 and Ki67 is highlighted by the white square. **B** overlay, **C** insulin, **D** PDX1, **E** Ki67. **F**–**I** Representative image of a cell positive for glucagon, ARX and Ki67 is highlighted by the white square. **F** overlay, **G** glucagon, **H** ARX, **I** Ki67. Scale bar = 5 µm (Color figure online)

Ki67-positive extra-islet endocrine cells were present in all age groups (0–2% of the 200 annotated cells in each donor, Fig. 4A), with no significant difference between groups (p = 0.8018). Only two extra-islet endocrine cells co-positive for insulin and Ki67 (Fig. 4B–E) were discovered; one was PDX1^+^ and present in a 5-year-old donor and one was PDX1^−^ and present in a 19-year-old donor. Both INS^+^Ki67^+^ cells were found in the intra-acinar compartment. Cells co-positive for glucagon and Ki67 (Fig. 4F-I) were found in all age groups. See supplementary table S3 for full details of Ki67-positive cells.

## Discussion

In the current study, extra-islet insulin- and glucagon-positive cells scattered in the exocrine compartment have been examined in pancreatic tissue samples obtained from heart-beating organ donors treated as intended for transplantation. The median density of extra-islet INS^+^ and GCG^+^ cells were 17.3 and 22.9 cells/mm^2^, respectively, which is approximately fivefold higher than the islet density reported previously [20]. The relatively constant frequency of pancreatic extra-islet endocrine cells during the first 25 years of life suggests that new cells are formed with increasing age, as the pancreas size is increased several-fold during the same time period [3, 21]. At least part of this increase is likely derived from replication of existing cells as both INS^+^ and GCG^+^ cells had ongoing mitotic activity, as shown by the presence of nuclear Ki67 in a small number of extra-islet cells. However, if some of the mitotic cells form aggregates of cells, i.e., new islets, this would contribute to a reduction of single cells or groups with ≤ 4 cells with increasing age. De novo formation of endocrine cells has been reported from pancreatic ductal epithelial cells [11], nonendocrine pancreatic endothelial cells [22], acinar cells [12, 13] and transdifferentiation of endocrine cells [23, 24], and one or more of these mechanisms likely also contributes to replenishing the population of extra-islet insulin- and glucagon-positive cells with increasing age.

In the current study, we focused on intra-acinar extra-islet endocrine cells, and not on cells present within the ductal epithelium as neogenesis of beta-cells from ducts has been previously reported in several studies [19, 25, 26]. Although the Ki67^+^ cells constituted only 0–2% of the extra-islet endocrine cells, it is important to acknowledge the fact that the section analysed only represents a snapshot. Even with a labelling index of only 0.1%, meaning that one out of 1000 extra-islet endocrine cells are in active mitosis at a given time point, this would correspond to a doubling of the cell population within a 3-year period [9, 27]. Moreover, the number of replicating endocrine cells in the pancreas is likely underestimated due to methodological factors [7]. However, although Ki67-positive cells were discovered, due to the low number of these cells observed in this study, further investigation is required to clarify their role. Taken together, the examined extra-islet cells may have a role in increasing endocrine cell mass in the human pancreas which motivates further examination.

The majority of the Ki67-positive cells were glucagon- rather than insulin-positive and the median ratio of INS^+^ to GCG^+^ cells was 0.9 which is lower compared to the proportion within islets. Interestingly, many of the extra-islet INS^+^ and GCG^+^ cells in all age groups lacked the expression of both PDX1 and ARX, in islets this was a much rarer event. PDX1 and ARX are transcription factors required for the generation and maintenance of beta- and alpha-cells respectively [23]. The development and maintenance of the endocrine cells is intricate and involves a number of transcription factors, but this could indicate that several of the extra-islet cells are immature, newly formed and/or plastic. Furthermore, differentiation of glucagon-positive to insulin-positive cells, or vice versa, has been reported in animal studies, in vitro studies, and partial alpha-to-beta differentiation in human islets ex vivo [23]. It is feasible that de novo formation of insulin- and glucagon-positive cells from e.g. ducts [18, 26], followed by replication and further differentiation of the cells is a possible mechanism contributing to islet formation and replenishment during the first 25 years of life. However, ongoing differentiation cannot be examined with the methods used in this study and hence requires further investigations.

There are some limitations in this study. The extra-islet cells were only examined in 2-D, i.e. only one thin layer of the cells was visible in the analysed image, possibly leading to false positive annotation of co-localization. Therefore, the presence of some very rare cell types should be interpreted with caution. Additionally, only one section was examined from each tissue sample, theoretically increasing the risk that some annotated single cells represent the mantle of an islet. However, when Bouwens and Pipeleers studied units of ≤ 3 beta-cells, consecutive sections confirmed that the analysed single-cells were not part of larger islets. Therefore this risk was not considered sufficient to affect the conclusions of this study [18].

In summary, both the extra-islet insulin- and glucagon-positive cells were frequent compared to what has been reported for islets. The relatively constant frequency of pancreatic extra-islet endocrine cells suggests that new cells are formed with increasing age. At least part of this increase could be derived from replication of existing extra-islet endocrine cells as some with mitotic activity were discovered, but de novo formation of extra-islet endocrine cells likely also contributes to replenishing this population. Interestingly, a large proportion of the extra-islet endocrine cells lacked the expression of both PDX1 and ARX, which could indicate that the cells are immature, newly formed and/or plastic. This in combination with the mitotic activity also suggests that the cells could play an important role in expansion of beta-cell mass in situations of increasing demand, or in turnover of the endocrine cell population.

## Supplementary Information

Below is the link to the electronic supplementary material.

Supplementary file1 (PDF 636 kb)
