# Supplementary material for: Extra-islet cells expressing insulin or glucagon in the pancreas of young organ donors
Source: Acta Diabetol. 2024 Jun 18;61(9):1195–203. doi: 10.1007/s00592-024-02295-0 (PMC11379743; doi:10.1007/s00592-024-02295-0)
Supplement: Supplementary file 1 — Supplementary file1 (PDF 636 kb) [file 592_2024_2295_MOESM1_ESM.pdf]

Supplementary

Autostainer protocol. The staining protocol, including deparaffinisation, was run on the Autostainer BOND RX System from Leica Biosystems (21.2821). The protocol for the autostainer is found below.

1. Bond Dewax Solution (Leica, AR9222) x 3 at 72°C
2. Reagent Grade Alcohol (≥90 %) x 3
3. Bond Wash Solution (Leica, AR9590) x 5
4. Epitope Retrieval Solution 1 (ER1, pH 6, Leica, AR9961) for 20 min at 95°C (not used for antibody 1)
5. Bond Wash Solution x 3
6. PKI Blocking Buffer (Akoya Biosciences, NEL821001KT) for 5 min at RT
7. Primary Antibody, incubation time 30 min (Ab 2, 3 and 5) or 40 min (Ab 1 and 4)
8. Bond Wash Solution x 3
9. Secondary Antibody, HRP conjugated, incubation time 10 min
10. Bond Wash Solution x 5
11. Opal Fluorophore, incubation time 10 min
12. Bond Wash Solution x 4

Repeat steps 4-12, staining the next antibody, until all 5 antibodies are done.

13. Real Dapi (Akoya Biosciences, NEL821001KT) for 5 min
14. Bond Wash Solution x 4

Supplementary table 1 Primary antibodies, secondary antibodies, and Opals used for multiplex staining and immunofluorescence

Antibody/OPAL	Concentration	Diluted in	Catalog #	Company
**Insulin**	Ready-to-use	-	IR00261-2	Agilent
**PDX1**	5 µg/ml	Antibody diluent/Block	AF2419	R&D systems
**Glucagon**	1:500	Antibody diluent/Block	AC-0074 (EP-74)	Epitomics
**ARX**	5 µg/ml	Antibody diluent/Block	AF7068	R&D systems
**Ki67**	1:50	Antibody diluent/Block	M724029-2 (MIB-1)	Agilent
**Impress Rabbit**	Ready-to-use	Antibody diluent/Block	K4003	Dako
**ImPress Mouse**	Ready-to-use	Antibody diluent/Block	K4001	Dako
**Goat Sec**	1:2000	Antibody diluent/Block	Ab6885	Abcam
**Sheep Sec**	1:2000	Antibody diluent/Block	Ab6900	Abcam
**OPAL 480**	1:200	Amplification diluent	NEL821001KT	Akoya Biosciences
**OPAL 520**	1:200	Amplification diluent	NEL821001KT	Akoya Biosciences
**OPAL 570**	1:200	Amplification diluent	NEL821001KT	Akoya Biosciences
**OPAL 620**	1:150	Amplification diluent	NEL821001KT	Akoya Biosciences
**OPAL 690**	1:150	Amplification diluent	NEL821001KT	Akoya Biosciences
**AF647 goat-anti-guinea pig**	1:400	TBST	AB150187	Abcam
**AF488 rabbit-anti-goat**	1:400	TBST	A11078	Invitrogen
**AF647 donkey-anti-sheep**	1:400	5 % donkey serum	AB150179	Abcam
**AF488 donkey-anti-rabbit**	1:400	5 % donkey serum	A21206	Invitrogen

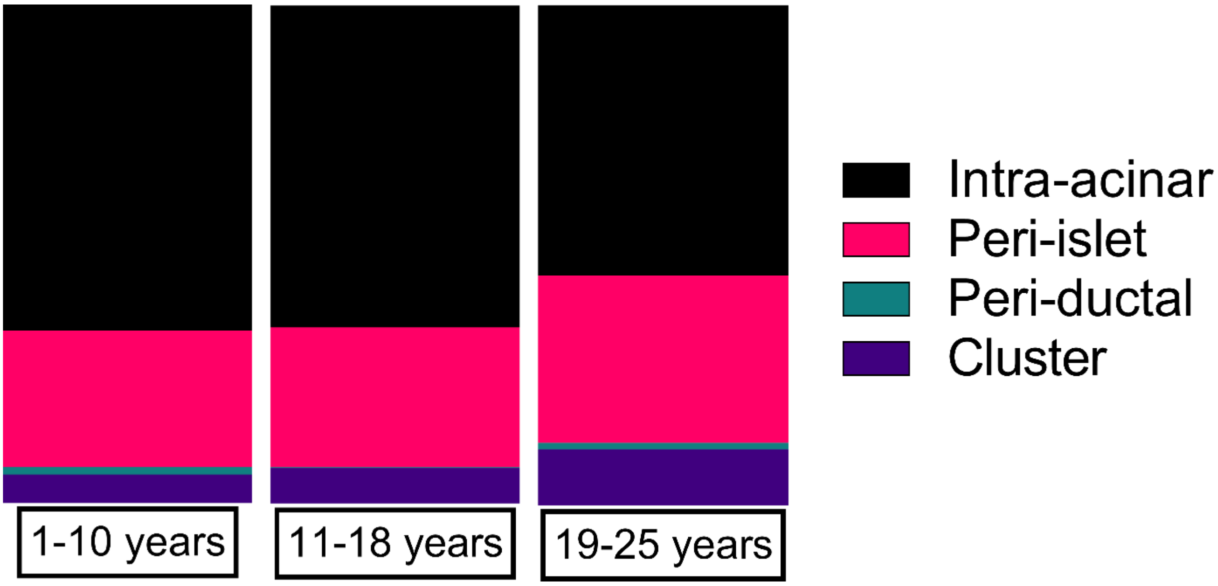

Supplementary Fig. 1 The mean proportions of the localisation of the cells in the different age groups

Intra-acinar: surrounded by acinar tissue and not being defined as peri-islet or peri-ductal. Peri-islet: being close to an islet (distance 0-3 cells) but outside of the islet perimeter. Peri-ductal: being close to a duct (distance 0-3 cells from larger ducts that were morphologically distinguishable within the exocrine parenchyma) but not within the ductal epithelium. Cluster: several single cells and/or groups of 2-4 cells located close together in a cluster.

Supplementary table 2 The proportion of extra-islet cell phenotypes in each donor 200 cells were annotated in each donor. Except for cells only positive for ARX, or co-positive for only PDX1 and ARX, the proportion (%) of all remaining phenotypes based on combinations of insulin, glucagon, PDX1 and ARX in each donor is illustrated. Total no of cells = when cells only positive for ARX, or co-positive for only PDX1 and ARX have been subtracted.

Donor No.	**GCG+**INS- PDX1-ARX-	**INS+**GCG- PDX1-ARX-	**GCG+**INS- **ARX+**PDX1-	**INS+**GCG- **PDX1+**ARX-	**GCG+**INS- **PDX1+**ARX-	**INS+**GCG- PDX1-**ARX+**	**GCG+**INS- **PDX1+ARX+**	**INS+**GCG- **PDX1+ARX+**	**INS+GCG+** PDX1-ARX-	**INS+GCG+** **PDX1+**ARX-	**INS+GCG+** PDX1-**ARX+**	**INS+GCG+** **PDX1+ARX+**	Total no of cells
A1	35	24	12	22	3	0	1	0	1	1	1	1	187
A2	22	42	12	19	2	0	0	0	3	0	0	0	196
A3	21	25	23	21	3	1	1	2	1	1	1	1	182
A4	44	15	19	15	3	1	1	0	2	0	1	0	188
A5	37	29	9	22	2	0	0	0	0	1	0	0	170
A6	20	33	23	17	3	1	2	0	1	0	1	0	182
B1	44	23	21	8	2	0	0	1	1	1	1	0	195
B2	29	27	15	25	1	0	0	1	2	1	0	0	181
B3	46	22	13	15	2	0	0	0	2	0	0	0	196
B4	24	28	32	14	1	1	0	0	0	0	1	0	165
B5	18	40	12	28	1	1	0	0	1	0	1	0	181
B6	13	37	24	21	2	0	0	0	2	0	1	0	188
B7	11	25	29	23	2	3	2	2	2	1	1	1	177
C1	43	22	20	9	4	1	0	0	2	0	1	0	196
C2	27	32	14	13	2	8	1	3	2	0	0	0	192
C3	45	30	7	12	3	0	0	0	4	0	0	0	194
C4	28	20	23	27	2	1	0	0	0	0	0	0	199
C5	49	16	12	17	3	0	0	1	1	1	0	0	186
C6	48	23	9	17	1	0	0	0	1	1	0	0	196
C7	41	38	4	10	2	1	0	1	3	0	1	0	186
C8	75	9	11	2	0	0	0	0	3	0	1	0	194

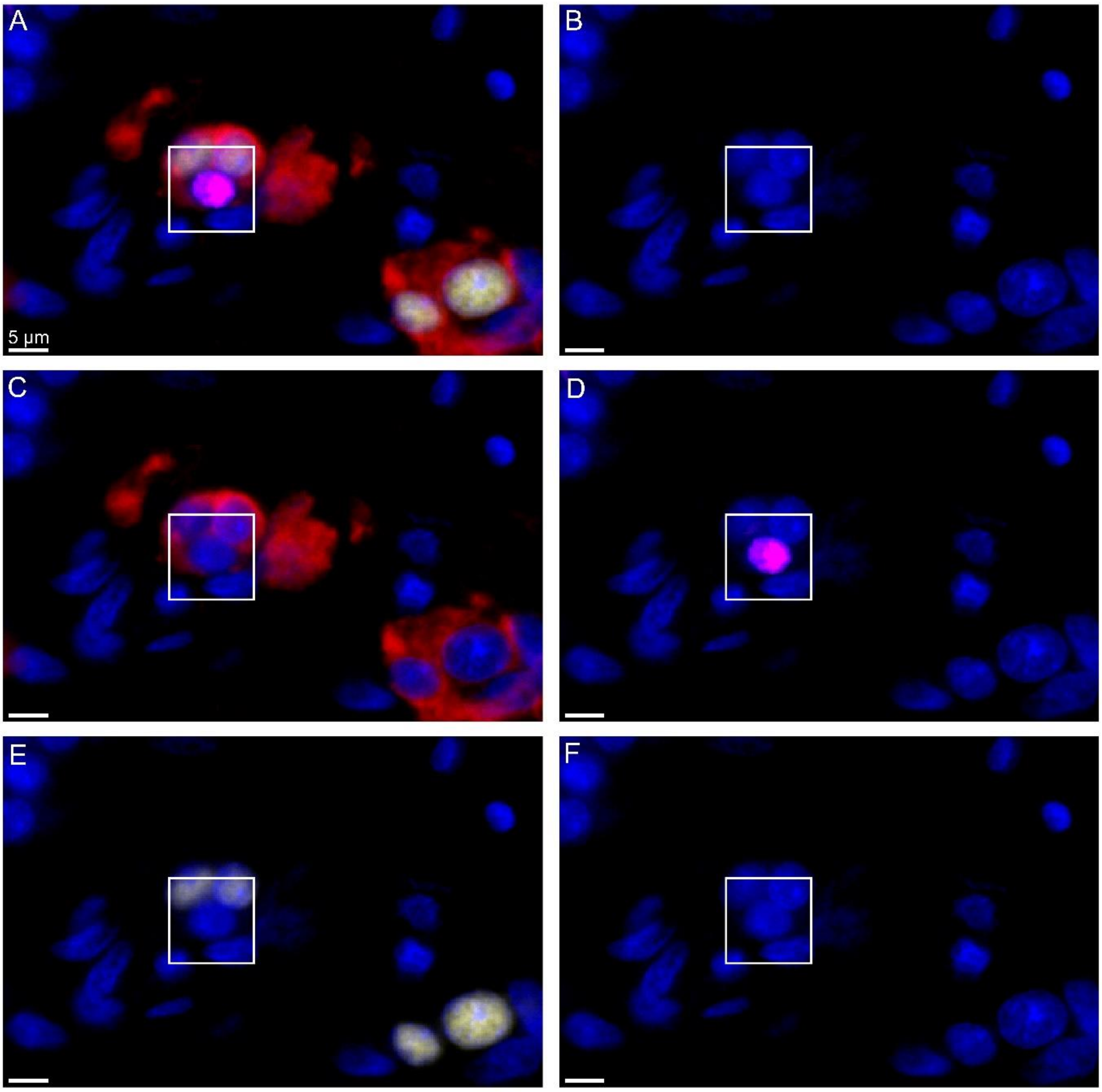

Supplementary Fig. 2 Representative images of a rare cell type

A-F) Endocrine cells were stained for insulin (green), glucagon (red), PDX1 (magenta), ARX (yellow), Ki67 (white) and nuclei (DAPI, blue). A representative image of a cell positive for glucagon and PDX1 is highlighted by the white square. A: overlay, B: nuclei, C: glucagon, D: PDX1, E: ARX, F: Ki67 (not expressed by this cell). Scale bar = 5 µm.

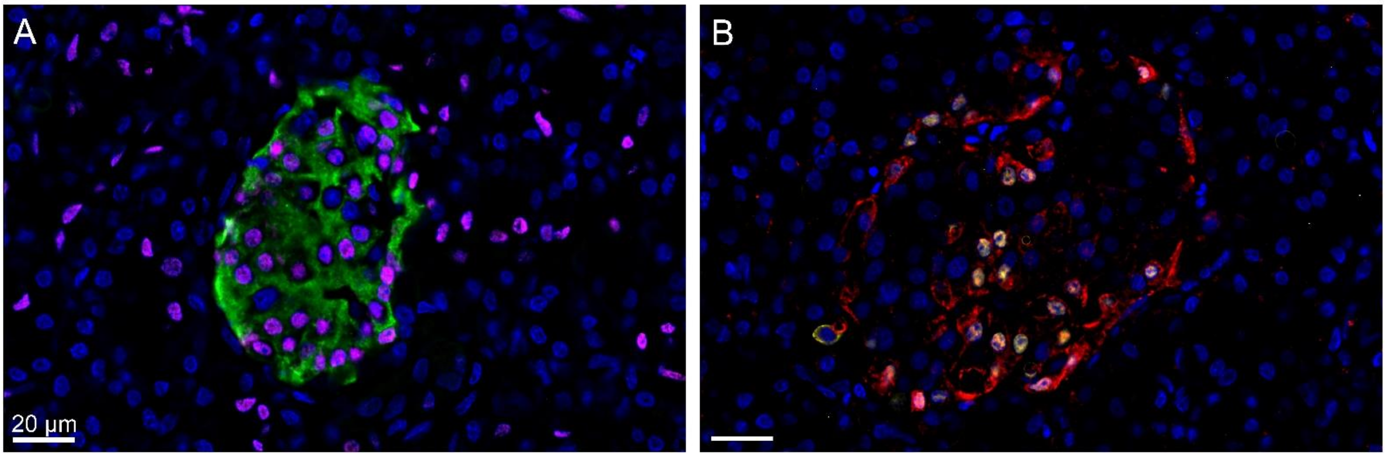

Supplementary Fig. 3 Representative images of islets

A) Immunofluorescent staining of insulin (green), PDX1 (magenta) and nuclei (DAPI, blue).

B) Immunofluorescent staining of glucagon (red), ARX (yellow) and nuclei (DAPI, blue). Scale bar = 20 μm.

Supplementary table 3. The characteristics of the Ki67-positive endocrine cells. Donor No.: Donor number, A (0-10 years old), B (11-18 years old), C (11-19 years old). Group: single cells as well as small groups of up to 4 cells were annotated, the number of cells in this specific group (1, 2, 3 or 4 cells) is displayed. Localisation: the localisation of the cells was defined as: 1) peri-islet: being close to an islet (distance 0-3 cells) but outside of the islet perimeter, 2) peri-ductal: being close to a duct (distance 0-3 cells) but not within the ductal epithelium, and 3) Intra-acinar: surrounded by acinar tissue and not being defined as peri-islet or peri-ductal. Cluster: several single cells and/or groups of 2-4 cells located close together in a cluster. Phenotype: markers expressed other than Ki67 (GCG=glucagon, INS=insulin).

Donor No.	No. of cells in group	Localisation	Phenotype	
A1	-	-	-	-
A2	-	-	-	-
A3	1	Intra-acinar	INS+	PDX1+
A3	1	Intra-acinar	GCG+	ARX+
A4	2	Peri-islet	GCG+	-
A5	-	-	-	-
A6		-	-	-
B1	2	Intra-acinar	GCG+	-
B1	1	Intra-acinar	GCG+	ARX+
B1	1	Intra-acinar	GCG+	ARX+
B1	1	Intra-acinar	GCG+	ARX+
B2	1	Peri-islet	GCG+	-
B3	-	-	-	-
B4	-	-	-	-
B5	-	-	-	-
B6	1	Intra-acinar	GCG+	PDX1+
B7		-	-	-
C1	2	Intra-acinar	GCG+	-
C1	1	Peri-islet	GCG+	-
C1	2	Intra-acinar	INS+	-
C2	1	Intra-acinar	GCG+	ARX+
C2	1	Intra-acinar	GCG+	ARX+
C2	3	Intra-acinar	GCG+	ARX+
C3	-	-	-	-
C4	-	-	-	-
C5	-	-	-	-
C6	1	Cluster	GCG+	-
C7	1	Peri-islet	GCG+	-
C8	-	-	-	-
